# Clinical determinants of social media use in individuals with schizophrenia

**DOI:** 10.1371/journal.pone.0225370

**Published:** 2019-11-20

**Authors:** Gurpreet Rekhi, Mei San Ang, Jimmy Lee

**Affiliations:** 1 Research Division, Institute of Mental Health, Singapore, Singapore; 2 North Region & Department of Psychosis, Institute of Mental Health, Singapore, Singapore; 3 Lee Kong Chian School of Medicine, Nanyang Technological University, Singapore, Singapore; Harvard University, UNITED STATES

## Abstract

This study aimed to examine the prevalence of social media use and its association with symptoms in individuals with schizophrenia. 265 individuals with schizophrenia were assessed. Symptoms were assessed on the Positive and Negative Syndrome Scale (PANSS) and the Clinical Assessment Interview for Negative Symptoms (CAINS). Information on social media use was collected. Logistic regressions were used to explore the association between social media use and socio-demographic and clinical characteristics of the participants. Of the 265 study participants, 139 (52.5%) used social media in the last week. Fifty-six (21.1%) of the study participants used more than one social media site in the last week. Facebook was the most popular social media site. Age, highest education level, monthly household income, PANSS negative and depression factor scores were significantly associated with social media use. Amongst negative symptoms, the CAINS motivation-pleasure (MAP) social factor scores were found to be significantly associated with social media use. Our study results suggested that the assessment of social interactions via social media should be considered in the clinical assessment of individuals with schizophrenia. Secondly, our results suggested that the development of treatment programs supported by social media platforms may be useful for certain groups of individuals with schizophrenia. Younger patients with above secondary level education, higher family income and lower symptom severity are likely to be avid users of social media and would be suitable candidates to receive illness related information or clinical interventions via social media.

## Introduction

The use of social media has witnessed a surge globally over the past two decades. It is increasingly being used to connect with others, share information, provide entertainment and receive the latest news. It was estimated that 72% of the American population used at least one social media platform in early 2019 [1, 2]. In the local Singapore population, rates of social media use were reported to be slightly higher for the same period [2]. Social media use was reported to be higher among younger individuals and those with higher education and higher income [1].

Similar to their peers, 33% to 71% of individuals with mental illnesses use social media [3–6], to share information and interests, to blog and make friends, as well as to discuss and seek advice for their symptoms [7, 8]. The rate of social media use was reported to be significantly higher in younger patients aged 18–29 years, as compared to older patients [6]. Studies conducted on adolescents and young adults with mental illnesses reported that almost all of them regularly used social media, with an average of 2.6 hours daily [7]. Social media use was reported to be independent of psychological well-being or psychiatric symptoms in individuals with serious mental illnesses [4]. The additional benefit of social media is that it provides a non-threatening and less anxiety provoking environment, which may be the reason individuals with mental illness would be comfortable to use it to interact socially [9]. Thus, social media can be harnessed to provide therapeutic interventions to individuals with mental illness like schizophrenia [10]. It provides mental health clinicians a platform to reach out and engage with patients, especially youths in their earliest stages of illness, which could have beneficial effect on the progression of the illness [7, 10].

There is insufficient data on the prevalence of social media use and factors that influence its use in individuals with schizophrenia. It was reported that around half (47%) of individuals with schizophrenia used social media, and around a quarter of them used social media networking (SMN) sites daily [11]. Facebook was reported to be the most popular social media site among social media users in the same study [11]. More than half of the current social media users with schizophrenia reported that social media websites helped them to interact with family and friends, as well as to socialize more with people outside their homes [11].

Symptoms of schizophrenia, e.g. social withdrawal and avolition, can have an impact on social media use, similar to their impact on other areas of life in individuals with schizophrenia [12, 13]. However, evidence on demographic or clinical factors associated with social media use in schizophrenia is scarce. This knowledge is needed to help clinicians understand the profile of patients most likely to engage with them through social media. Therefore, the present study aimed to examine the prevalence of social media use in individuals with schizophrenia, and to examine the association of severity of symptoms in schizophrenia with social media use. We hypothesized that about half of our study participants would be using social media. We also hypothesized that the likelihood of social media use would be negatively associated with the severity of symptoms in schizophrenia.

## Methods

### Setting and study participants

Study participants were recruited from the outpatient clinics at the Institute of Mental Health (IMH), Singapore. English-speaking patients with a diagnosis of schizophrenia, between the ages of 21 to 65 years, were included in the study. Those with (i) a current alcohol or substance use disorder, (ii) mental retardation (IQ<70), or (iii) a history of head injury or neurological disorder were excluded. A total of 277 participants were recruited. Three out of the 277 participants withdrew from the study, and from the remaining sample, 9 participants were not able to provide information on monthly household income (data required for analyses), and were therefore excluded from the study. Thus, the sample reported is 265. Ethics approval for this study was provided by the National Healthcare Group's Domain Specific Review Board, Singapore. Written informed consent was obtained from all the participants.

### Assessments

Socio-demographic information was collected from all participants. The Structured Clinical Interview for DSM-IV (SCID-I) [14] was used to ascertain the diagnosis of schizophrenia. Severity of schizophrenia symptoms was assessed on the Positive and Negative Syndrome Scale (PANSS) [15]. The PANSS has 30 items rated on a Likert scale from 1 (absent) to 7 (extreme); a higher rating on the PANSS indicates higher severity of symptoms. These items assess the positive (7 items), negative (7 items) and general psychopathology (16 items) domains of schizophrenia. The PANSS was validated for use in the local population, with a 5-factor structure, namely, positive factor, negative factor, excitement factor, depression factor, and cognitive factor [16]. The PANSS factor scores were computed by summation method.

The Clinical Assessment Interview for Negative Symptoms (CAINS) [17–19] was used to measure the severity of negative symptoms. The CAINS consists of two subscales, motivation-pleasure (MAP) subscale (items 1–9) and an expression (EXP) subscale (items 10–13). The MAP items measure individuals’ behaviors, motivation and pleasure related to social, occupational, and recreational activities; the EXP items assess facial expression, expressive gestures, vocal expression, as well as quantity of speech. Each item is rated on a 5-point scale, ranging from absent (0) to severe (4). A 4-factor structure of the CAINS has been validated for use in the local population; the 4 factors being: MAP social, MAP vocational, MAP recreational and EXP [20]. The ratings on the PANSS and CAINS were based on symptoms in the last week. The CAINS factor scores were computed by summation method. To evaluate the severity of each negative symptom factor, an average factor score was computed by dividing the sum of item ratings with the number of items in each factor.

Participants were asked the following questions with regard to their social media use in the week before the assessment:

1. Did you use social media in the last week?
2. Which social media sites did you access in the last week?
3. How frequent did you use social media in the last week?

In the data analyses, social media use in the last week was coded as 0 = No and 1 = Yes. Similarly, the use of each social media site in the last week (Facebook, YouTube, WhatsApp, Instagram, Twitter and others) was coded as 0 = No and 1 = Yes. The frequency of social media use in the last week was coded as follows: 1 = Once or more daily in the last week, 2 = 3–6 times/last week, 3 = 1–2 times/last week and 4 = Less than once/last week.

Three raters conducted the study assessments: a research clinician, a master level research psychologist and a bachelor level research psychologist. All raters had at least 2 years’ research experience related to schizophrenia. All raters were trained in the administration and scoring of the rating scales used in the study. The raters obtained an intra-class correlation coefficient (ICC) of above 0.80 for ratings on the PANSS and the CAINS. Interrater agreement was maintained by regular meetings between the raters.

### Statistical analyses

All statistical analyses were performed using IBM SPSS Statistics 23. Mean and standard deviations or median (if normality was not satisfied) were calculated for continuous variables,–whereas frequencies and percentages were calculated for categorical variables. Logistic regressions were used to investigate the association between likelihood of social media use and severity of symptoms. The estimates obtained from these analyses were adjusted Odds Ratios (OR). An adjusted OR > 1 meant a positive association between the independent variable (IV) and the dependent variable (DV), whereas an adjusted OR<1 meant a negative association between the IV and the DV. In the first logistic regression model, the PANSS factor scores were entered as IVs and social media use in the last week as the DV. In the second model, the CAINS factor scores were used as IVs and social media use as DV. Age, gender, highest education level and monthly household income were entered as covariates in both of the models. Statistical significance was established at p < .05.

## Results

### Socio-demographic characteristics of the study sample

Socio-demographic characteristics of the study participants are shown in Table 1. Mean age of the sample was 40.38 (SD = 10.26) years. The sample consisted of 148 males (55.8%) and 117 females (44.2%). Most of the participants were of Chinese ethnicity (n = 223, 84.2%). Thirty-five (13.2%) participants were currently married, 206 (77.7%) were never married, 20 (7.5%) were divorced, 2 (0.8%) were separated and 2 (0.8%) were widowed. Most of the participants (n = 165, 62.3%) were educated above secondary school level. Median monthly household income was Singapore dollars 2000.00 (IQR 650.00–4200.00).

**Table 1:** Socio-demographic characteristics of the study participants and rates of social media use across socio-demographic groups.

	n (%)	Participants using social media, n (%)
Age (years)		
21–30	51 (19.24)	40 (78.4)
31–40	84 (31.70)	51 (60.7)
41–50	79 (29.81)	38 (48.1)
Above 50	51 (19.24)	10 (19.6)
Gender		
Males	148 (55.8)	72 (48.6)
Females	117 (44.2)	67 (57.3)
Ethnicity		
Chinese	223 (84.2)	122 (54.7)
Malay	20 (7.5)	9 (45)
Indians	21 (7.9)	7 (33.3)
Others	1 (0.4)	1 (100)
Marital status		
Currently Married	35 (13.2)	25 (71.4)
Never married	206 (77.7)	104 (50.5)
Divorced	20 (7.5)	9 (45)
Separated	2 (0.8)	0 (0)
Widowed	2 (0.8)	1 (50.0)
Highest education level		
Secondary and below	100 (37.7)	38 (38.0)
Above secondary	165 (62.3)	101 (61.2)
Monthly household income (SGD)		
0–625.00	67 (25.3)	18 (26.9)
626.00–2000.00	71 (26.8)	35 (49.3)
2001.00–4200.00	62 (23.4)	38 (61.3)
Above 4200.00	65 (24.5)	48 (73.8)

SGD, Singapore dollars

### Clinical characteristics of the study sample

The clinical characteristics of the study participants are shown in Table 2. The PANSS total ranged from 32 to 108. Mean PANSS total score of 57.90 (SD = 12.67) suggested that the study participants had mild to moderate severity of illness [21]. The CAINS total ranged from 0 to 37. The average factor scores for CAINS MAP social, CAINS MAP vocational, CAINS MAP recreational and CAINS EXP factors were 1.19 (SD = 0.85), 1.92 (SD = 1.28), 1.13 (0.81) and 1.18 (SD = 0.94) respectively, suggesting that the participants had mild to moderate severity of symptoms in all of the 4 negative symptom domains.

**Table 2:** Clinical characteristics of the study participants.

	*Mean*	*S*.*D*.
Positive and Negative Syndrome Scale		
Positive factor	8.24	4.28
Negative factor	11.20	4.01
Excitement factor	4.58	2.02
Depression factor	5.62	2.50
Cognitive factor	4.33	1.63
Total score	57.90	12.67
Clinical Assessment Interview for Negative Symptoms		
MAP social factor	4.74	3.40
MAP vocational factor	3.84	2.57
MAP recreational factor	3.40	2.43
Expression factor	4.72	3.74
Total score	16.70	7.87

MAP, Motivation-Pleasure

### Prevalence and patterns of social media use

Almost half of the study participants (n = 139, 52.5%) used social media in the week before the research assessment. Table 1 shows the rates of social media use across the socio-demographic groups. Use of social media differed across the various socio-demographic groups. Number of social media users were highest among participants aged 21–30 years old (n = 40, 78.4%), females (n = 67, 57.3%), Chinese (n = 122, 54.7%), currently married (n = 25, 71.4%), educated above secondary level (n = 101, 61.2%), and those in the highest monthly household income group (n = 48, 73.8%).

**Fig 1:**
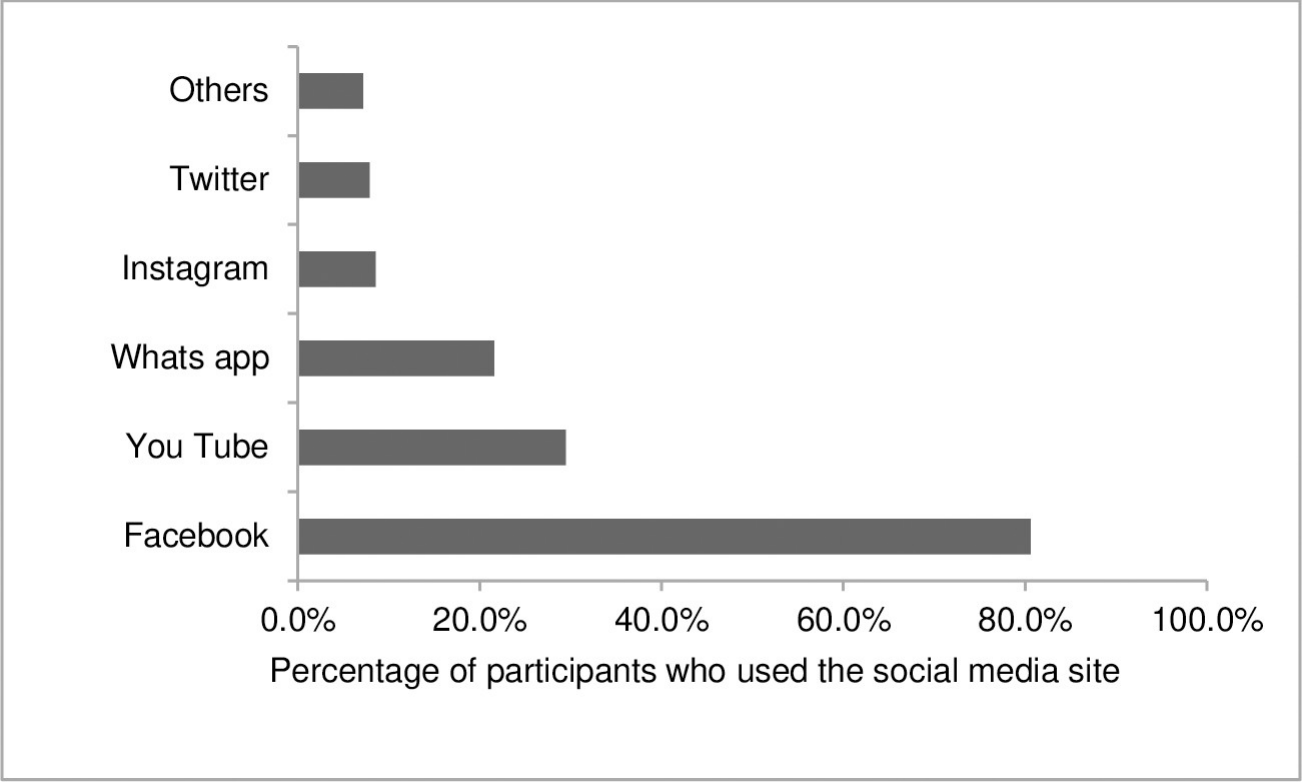
Social media sites visited in the last week by study participants who used social media (n = 139) Others: Yahoo, WeChat, Viber, Skype, LinkedIn, Line, Blogger and Unspecified Chat group.

More than half (n = 83, 59.7%) of those who used social media used only 1 social media site, 40 (28.8%) accessed 2 sites and 16 (11.5%) used more than 2 social media sites in the last week. Facebook was the most popular social media site (n = 112, 80.6%) followed by YouTube (n = 41, 29.5%) and WhatsApp (n = 30, 21.6%) (Fig 1). 42.3% and 15.5% of the entire sample accessed Facebook and YouTube respectively in the last week.

Data on frequency of social media use was missing for 23 participants. Of the remaining 116 participants, 73 (62.9%) used social media daily in the last week, 18 (15.5%) used it 3–6 times in the last week, 21 (18.1%) used it 1–2 times, whereas 4 (3.4%) reported using social media less than once in a week.

### Association of severity of symptoms with social media use

Table 3 shows the results of the first regression analysis. PANSS negative (OR = 0.915, 95% CI: 0.845–0.993; p<0.05) and PANSS depression factor scores (OR = 0.866, 95% CI: 0.759–0.989; p<0.05) were significantly and negatively associated with social media use. Age (OR = 0.918, 95% CI: 0.890–0.947; p<0.001) was significantly and negatively associated with social media use, whereas highest education level (OR = 1.849, 95% CI: 1.013–3.375; p<0.05) and monthly household income (OR = 1.251, 95% CI: 1.099–1.424; p<0.05) were significantly and positively associated with social media use. In the second regression model, CAINS MAP social was significantly and negatively associated with social media use (OR = 0.896, 95% CI: 0.812–0.988; p<0.05). Similar to the previous model, age (OR = 0.919, 95% CI: 0.890–0.948; p<0.001) was significantly and negatively associated with social media use, whereas highest education level (OR = 2.044, 95% CI: 1.123–3.719; p<0.05) and monthly household income (OR = 1.206, 95% CI: 1.062–1.370; p<0.05) were found to be significantly and positively associated with social media use (Table 4).

**Table 3:** Logistic regression to investigate association between social media use and PANSS factors.

	B	S.E.	Wald	p	O.R.	95% C.I. for O.R.	
						Lower	Upper
Age	-.086	.016	29.411	< .001	.918	.890	.947
Gender	.356	.302	1.386	.239	1.427	.789	2.579
Highest education level	.615	.307	4.013	.045	1.849	1.013	3.375
Monthly household income	.224	.066	11.458	.001	1.251	1.099	1.424
PANSS Positive factor	.057	.039	2.163	.141	1.059	.981	1.143
PANSS Negative factor	-.088	.041	4.519	.032	.915	.845	.993
PANSS excitement factor	-.014	.076	.035	.851	.986	.849	1.144
PANSS depression factor	-.144	.067	4.522	.033	.866	.759	.989
PANSS cognitive factor	-.163	.104	2.434	.119	.850	.692	1.043

PANSS, Positive and Negative Syndrome Scale

**Table 4:** Logistic regression to investigate association between social media use and negative symptom factors.

	B	S.E.	Wald	p	O.R.	95% C.I. for O.R.	
						Lower	Upper
Age	-.085	.016	27.375	< .001	.919	.890	.948
Gender	.183	.302	.370	.543	1.201	.665	2.170
Highest education level	.715	.305	5.475	.019	2.044	1.123	3.719
Monthly household income	.187	.065	8.284	.004	1.206	1.062	1.370
CAINS MAP social factor	-.110	.050	4.824	.028	.896	.812	.988
CAINS MAP vocational factor	-.045	.062	.534	.465	.956	.847	1.079
CAINS MAP recreational factor	-.096	.069	1.955	.162	.908	.794	1.039
CAINS Expression factor	-.076	.040	3.636	.057	.927	.857	1.002

CAINS, Clinical Assessment Interview for Negative Symptoms; MAP, Motivation-Pleasure

## Discussion

This study sought to examine the prevalence of social media use in individuals with schizophrenia, and to examine the association of symptoms in schizophrenia with social media use. The rate and frequency of social media use found in our study was comparable to a previous report on social media use in schizophrenia [11]. The most popular social media site among our participants was Facebook, which is also consistent with that reported in the same study [11].

Our study results suggested that the severity of negative symptoms, specifically a decrease in the CAINS MAP social factor scores, was associated with a higher likelihood of social media use. Reduction in drive or interest in forming close relationships with others is a core feature of schizophrenia [22, 23]. The social networks of these individuals are smaller and consisting mainly of family members or relatives, in contrast to healthy individuals who have larger and more complex social networks, consisting of a higher proportion of non-relatives [24, 25]. Further, individuals with schizophrenia have impairment in anticipation of pleasure (anhedonia) related to an activity, leading to decreased behaviours and efforts to perform that activity [23, 26]. Thus, it is possible that social interactions (both real life and virtual) are anticipated as less pleasurable or less rewarding, leading to less efforts to make social contacts both in real life and through social media.

Severity of depressive symptoms was also found to be related to social media use in our sample. There is a high prevalence of depressive symptoms in schizophrenia [27, 28]. Schizophrenia patients with depressive symptoms have poorer family and social relationships as compared to those without depressive symptoms [27]. Depressed individuals may pay more attention to negative social interactions, and may be more sensitive to both social rejection and acceptance [29]. These impairments may disincentive social interactions and further decrease their motivation to interact. Moreover, these real life impairments could affect virtual social interactions too, which could explain why severity of depressive symptoms was found to be associated with social media use in schizophrenia.

We also found that schizophrenia individuals who were younger and had above secondary level of education were more likely to use social media. This is consistent with previous reports that lower age is associated with more social media and internet use in individuals with mental illnesses [6, 8, 30]. Similarly, educational attainment is related to more internet use among these individuals [6], as well as, among those with schizophrenia [31]. This finding could also be a reflection of the trend seen in the general population, that is, social media use is more common in younger individuals and those with higher education [1]. Further, it is intuitive that higher education level would facilitate understanding and use of internet and devices like smartphones or computers, and therefore, might increase the likelihood of social media use.

Further, higher household income was associated with social media, similar to the trend reported in the general population [1]. Schizophrenia is associated with lower socioeconomic status [32–34]. Lower household income might affect the accessibility of internet and devices like smartphones or computers needed to access internet. This could explain the association between social media use and household income. Individuals with psychotic disorders have been reported to have lower income and less access to internet as compared to healthy controls [35].

This study is one of the few studies on the use of social media in individuals with schizophrenia. Moreover, to our knowledge, this is the first study exploring the association of schizophrenia symptom domains with social media use. Our study has important implications. Social media is becoming an increasingly popular platform for socializing, and our results suggested that the severity of symptoms in schizophrenia is negatively associated with its use. Therefore, it would be useful to consider the assessment of social interactions via social media in the clinical assessment of social functioning in individuals with schizophrenia. Secondly, previous reports suggested that individuals with psychosis are open to using social media to obtain help or advice from mental health clinicians for symptom management [7]. It was also reported that online social media integrated with evidence-based interventions could benefit individuals with psychosis [10]. Likewise, future studies are needed to evaluate the acceptability and therapeutic potential of social media in individuals with schizophrenia. Our results can potentially guide the development of treatment programs supported by social media platforms for individuals with schizophrenia. Younger patients with above secondary level education, higher family income and lower symptom severity are likely to be avid users of social media and would be suitable candidates to receive illness related information or clinical interventions via social media.

Our study has certain limitations. We did not use a specific questionnaire to assess social media use. Therefore, we did not have details on the quality of social media use and there was some missing data on frequency of its use. This information could provide further insight into the relationship between the social media use and symptoms of schizophrenia. Secondly, information collected on social media use was based on self-reports, which could be biased. Participants may give responses which are socially desirable (i.e. social desirability response bias), which could have led to an over-estimation of the participant reported rates of social media use in the current study [36]. Moreover, we did not have a control group to compare the rates and patterns of social media use. Future research with a control group and a comprehensive questionnaire can provide valuable insights in this area.

In conclusion, we found that around half of the individuals with schizophrenia used social media in the last week; those who were younger, educated above secondary school level, had higher household income and lower severity of negative and depressive symptoms were more likely to use social media. Our study provides preliminary data regarding social media use and its association with symptoms in individuals with schizophrenia. More research is needed in this area, to facilitate the future potential application of social media in the clinical management of individuals with schizophrenia.
